# The early transition to cold-induced browning in mouse subcutaneous white adipose tissue (scWAT) involves proteins related to nerve remodeling, cytoskeleton, mitochondria, and immune cells

**DOI:** 10.1080/21623945.2024.2428938

**Published:** 2024-12-06

**Authors:** Magdalena Blaszkiewicz, Cory P. Johnson, Jake W. Willows, Miranda L. Gardner, Dylan R. Taplin, Michael A. Freitas, Kristy L. Townsend

**Affiliations:** a Department of Neurological Surgery, The Ohio State University, Columbus, OH, USA; b Graduate School of Biomedical Science and Engineering, University of Maine, Orono, ME, USA; c Department of Cancer Biology and Genetics, Comprehensive Cancer Center, The Ohio State University, Columbus, OH, USA; d Ohio State Biochemistry Program, Chemistry and Biochemistry, The Ohio State University, Columbus, OH, USA; e School of Biology and Ecology, University of Maine, Orono, ME, USA; f Department of Chemical and Biomedical Engineering, University of Maine, Orono, ME, USA

**Keywords:** Adipose immune cell recruitment and activation, adipose remodelling, adipose tissue innervation, Mitochondria, Adipsoe proteomics

## Abstract

White adipose tissue (WAT) is a dynamic organ capable of remodelling in response to metabolic state. For example, in response to stimuli such as cold exposure, WAT can develop inducible brown adipocytes (‘browning’) capable of non-shivering thermogenesis, through concurrent changes to mitochondrial content and function. This is aided by increased neurite outgrowth and angiogenesis across the tissue, providing the needed neurovascular supply for uncoupling protein 1 activation. While several RNA-sequencing studies have been performed in WAT, including newer single cell and single nuclei studies, little work has been done to investigate changes to the adipose proteome, particularly during dynamic periods of tissue remodelling such as cold stimulation. Here, we conducted a comprehensive proteomic analysis of inguinal subcutaneous (sc) WAT during the initial ‘browning’ period of 24 or 72hrs of cold exposure in mice. We identified four significant pathways impacted by cold stimulation that are involved in tissue remodelling, which included mitochondrial function and metabolism, cytoskeletal remodelling, the immune response, and the nervous system. Taken together, we found that early changes in the proteome of WAT with cold stimulation predicted later structural and functional changes in the tissue that are important for tissue and whole-body remodelling to meet energetic and metabolic needs.

## Introduction

Obesity is tightly linked with increased risk for numerous metabolic disorders. Obesity is caused by an energy imbalance with more energy intake than expenditure, which increases an individual’s risk for developing cardiometabolic comorbidities such as cardiovascular disease, type 2 diabetes mellitus, peripheral neuropathy, and metabolic syndrome – all of which increase mortality. According to the National Heart, Lung, and Blood Institute, excess abdominal (or visceral) fat is one of the five top-risk factors for metabolic syndrome. By contrast, subcutaneous white adipose tissue (scWAT) is considered a healthier WAT depot due to its increased insulin sensitivity and protective immune and adipokine profiles. scWAT is a highly dynamic tissue that is constantly remodelling in response to energy demands. Weight loss of even 10% can lead to improvements in insulin resistance in obese individuals [1], underscoring how quickly adipose tissue can regain metabolic control. In fact, the ability of adipose tissues to expand is linked with the risk for metabolic disease by preventing ectopic lipid deposition in other tissues, thereby reducing lipotoxic free fatty acids in the circulation that are not stored properly in adipocyte lipid droplets. In general, a healthy adipose mass is strongly correlated with whole body metabolic health, at any BMI. In response to various stimuli, including situations of negative energy balance such as cold stimulation, the tissue undergoes a process of ‘browning’, during which new beige/brite adipocytes differentiate from precursor cells (or potentially also transdifferentiate from mature white adipocytes), and express uncoupling protein 1 (UCP1) in newly generated mitochondria, enabling non-shivering thermogenesis [2–5]. This ‘browning’ capability of WAT confers metabolic benefits including greater uptake of circulating glucose and lipids, giving activated brown/beige adipocytes numerous anti-obesity and anti-diabetes roles [6].

Cold exposure is a potent activator of the sympathetic nervous system (SNS) in adipose tissues (brown and white) as well as subsequent scWAT browning. Release of the SNS neurotransmitter norepinephrine acts on beta-adrenergic receptors on adipocytes and preadipocytes to stimulate browning, lipolysis and non-shivering thermogenesis via activation of UCP1 [7–10]. Importantly, the regions of browning in scWAT (‘beige islets’) are also areas of dense sympathetic innervation [11], underscoring the importance of sympathetic drive for these metabolically-demanding processes. Cold acclimation studies in mice have characterized changes in the adipose milieu that include adipocyte phenotype [7–10], immune cell polarization and recruitment [12–18], release of secreted lipokines [19–21], adipokine/BATokine profiles in the tissue [22–24], mitochondrial biogenesis [25–29], and nervous system modulation [8, 30–36]. Although these functional alterations have been, and are currently, active areas of research regarding adipose tissue plasticity and healthy expansion, very little is known about the molecular mechanisms that *mediate* the plasticity and remodelling dynamics during the process of cold-stimulated browning of mouse scWAT.

Big data ‘−omics’ research has become increasingly prevalent due to the power of high-throughput analyses of molecular changes at the transcriptional and translational levels, as well as metabolite/lipid analyses. The multi-omic revolution includes the use of standard proteomic analysis of adipose depots [37–40], although assessments of smaller peptides in the tissue (including neuropeptides, released from adipose sensory nerves [41]) remain a technical challenge in standard proteomic/peptidomic workflows. Work to date has identified proteomes that are unique to each adipose depot [42–44]. There have been many cold-related transcriptomic studies from microarray analyses [45–48], to bulk RNAseq and single cell/single nuclei RNAseq studies [49–53], but little has been done in the way of proteomic analysis of cold-stimulated scWAT. One study assessed the proteome of WAT and BAT in mice housed at room temperature (20°C) versus thermoneutrality (28°C) [54], and only one study that we are aware of has analysed the proteome of cold-stimulated (4°C) scWAT [55]. In that study, the authors identified a transcription factor, YBX1, which peaks in protein expression after 24 h of cold exposure and is required to initiate the browning program [55].

These data are critical for understanding the initiation of browning; however, we do not yet understand the orchestration of biological processes that mediate the transitional stages during adaptive thermogenesis and tissue remodelling in the early days of cold exposure. Therefore, we report proteomic profiling of inguinal scWAT (ing-scWAT) during this early transition to browning by comparing 24 hr and 72 hr of cold (5°C) exposure in mice, a critical period of tissue remodelling and plasticity. We used label-free mass spectrometry to quantify shifts in 2166 total proteins, and our dataset provides an essential resource for future investigation of the molecular mechanisms involved in scWAT browning and tissue remodelling pathways that precede structural and functional changes that impact whole body energetics and metabolism. Additionally, these data offer insights into the remodelling of several distinct cellular processes to facilitate cold adaptation, including shifts in protein expression related to mitochondrial biogenesis, the immune response, cytoskeletal remodelling, and vascular and nervous system modulation.

## Results

### Proteomic dataset generation and quality control

**Figure 1.**
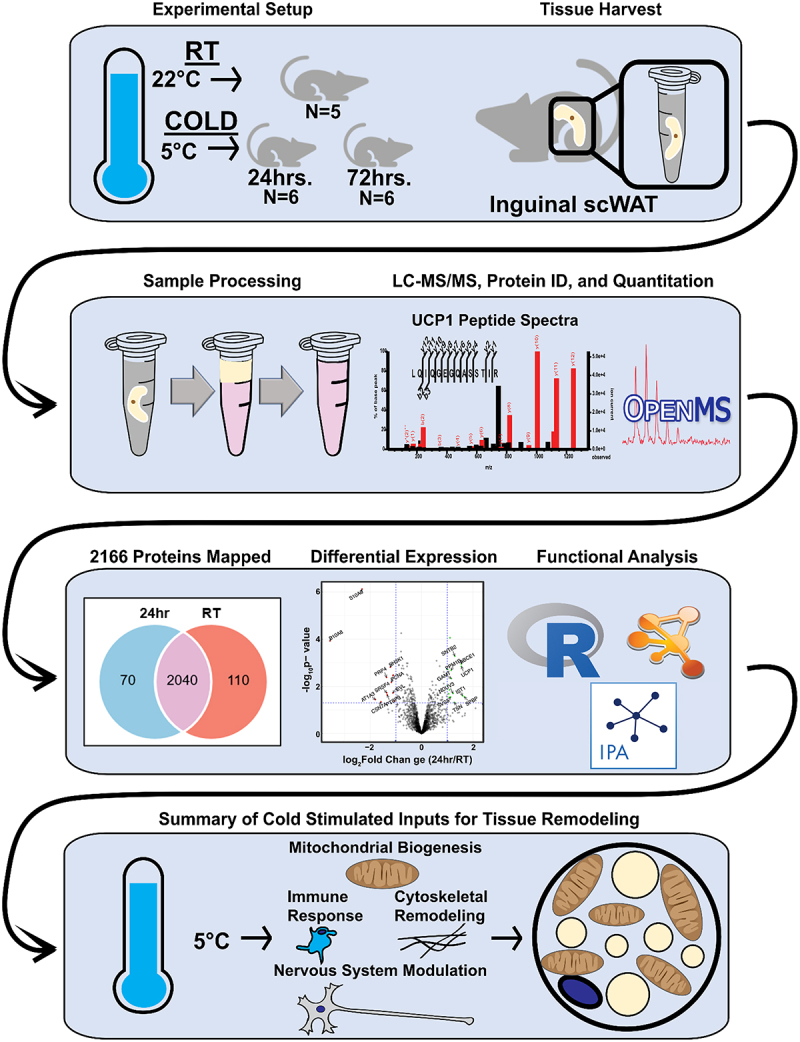
Schematic of experimental process.

We profiled the scWAT proteome in 12-week-old male C57BL/6 mice using the following three conditions: 1) room temperature (RT; *N* = 5 mice), 2) 24 hour (24 h) cold challenge (5°C) (*N* = 6 mice), and 3) 72 hour (72 h) cold challenge (*N* = 6 mice). Mice were randomly assigned to each group and sample size was decided upon based on our on our previous work. Our experimental workflow is presented in Figure 1. Researchers were blinded to groups immediately following tissue collection, prior to processing for proteomic assessment. Label-free proteomics datasets, while powerful, are also inherently prone to increased error rates when compared to labelled proteomic datasets. However, label-free approaches increase study-design flexibility and provide greater coverage of the proteome compared to labelled methods [56]. We initially assessed data and analysis quality using a newly developed and validated bioinformatic workflow to reduce the number of missing values and the false-discovery rate during protein identification [57], which was done by conducting principal components analysis (PCA) to assess variation across samples and treatment groups. We found little variation across treatment groups, though there was some variation among biological replicates in the 72 hr cold group. It is worth noting that one sample was the primary driver of variation among biological replicates at 72hrs cold, but post-normalization we found little difference between replicates (Figure S1A).

As mentioned previously, label-free abundance-based proteomics analysis has inherent errors, one of which is the need for value imputation when addressing the missing value problem [58]. Proper quantification of missing values is extremely important to yield confidence in assessing significance, and to ensure quality statistical analysis and avoid overinflation or underestimation of false-discovery rate (FDR). We observed that fewer than 400 proteins contained at least one missing value across the entire data set (*n* = 17); the majority of these 400 proteins were missing 3 or fewer values while a handful of proteins were missing 4 or more (Figure S1B). The ‘80% rule’ indicates that proteins with experimentally-determined values among at least 80% of samples have met the standard, allowing imputation for the rest of the samples. We found missing values in less than 25% of our dataset and few proteins that did not reach the missing value threshold (80% of 17 samples with experimental quantitation − 14 samples).

Finally, violin plots provided a secondary assessment of variation among biological replicates. We analysed the distribution of log_2_ intensity values among biological replicates, which provided a snapshot of protein intensity distribution among each sample. Samples before and after normalization did not change in intensity distribution (Figure S1C). Together, these results provided an ample assessment of data quality that prompted to further bioinformatic characterization.

### Early acclimation to cold challenge promoted a distinct shift in inguinal scWAT protein expression

**Figure 2.**
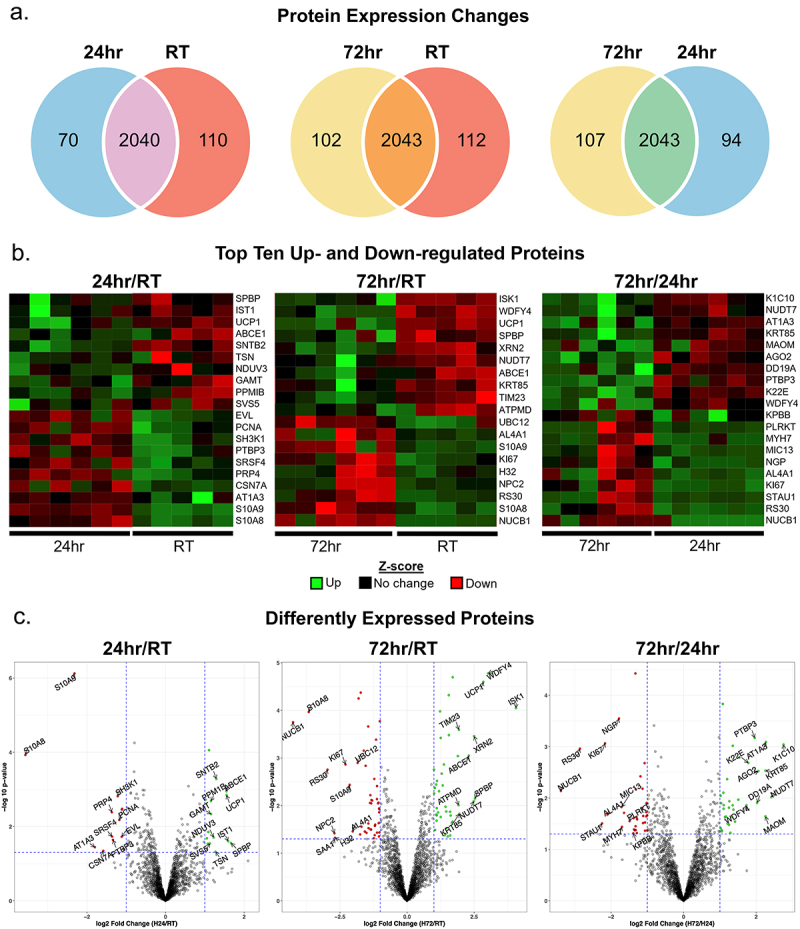
Differential expression analysis reveals shift in scWAT proteome. (a) Venn diagrams depicting protein profiling of adipose tissue with the non-significant proteins common to each group in the comparison (overlap of circle) and those proteins differentially expressed (left or right). (b) Heatmaps of 24hr/RT (left), 72hr/RT (middle), and 72hr/24h (right) pairwise comparisons that display upregulated (green) and downregulated (red) or no change (black) in protein expression per biological replicate. (c) Volcano plots were constructed for 24hr/RT (left), 72hr/RT (middle), and 72hr/24h (right) pairwise comparisons with log2 fold change and–log of p-value for significance. Upregulated (green) and downregulated (red) proteins are designated as such meeting the following criteria: fold change greater than 1.2 or −1.2 respectively and a *p*-value of ≤0.05 (1.3 –log). The top 20 most significant differentially expressed proteins (10 upregulated, 10 downregulated) are labelled (arrows).

Upon completion of our quality control screen, we investigated the data for differentially expressed (DE) proteins. In total, we quantified 2166 proteins, and then computed three pairwise comparisons −24 hr vs. RT (24 hr/RT) (Suppl. Table ST1), 72 h vs. RT (72 hr/RT) (Suppl. Table ST2), and 72 hr vs. 24 hr (72 hr/24 hr) (Suppl. Table ST1). Venn diagrams provided a simple yet informative visualization of differences in the number of DE proteins (Figure 2a). We found 70 DE proteins in animals exposed to 24 hr cold compared to RT, 102 significant DE proteins in animals exposed to 72 hr cold when compared to RT, 94 DE proteins in animals exposed to 24 hr cold compared to 72 hr cold, 107 DE proteins in animals exposed to 72 hr cold compared to 24 hr, 112 DE proteins expressed in RT when compared to 72 h cold animals, and 110 DE proteins expressed in RT animals when compared to those exposed to 24 hr cold.

In addition to the ‘birds-eye’ view obtained from Venn diagrams of large datasets, heatmaps aid in visualization of expression differences across biological replicates for all DE proteins, as well as subsets of DE proteins (Figure 2b). To better understand patterns and trends in DE proteins and insight into biological meaning of the adipose proteome during tissue remodelling, we isolated the top 20 DE proteins – the top 10 most upregulated and 10 most downregulated – from each pairwise comparison (Figure 2b). We next characterized the function of the proteins with the top largest and smallest logFC from each pairwise comparison (Suppl. Table ST4 and Suppl. Table ST5).

Overall, we saw little variation among biological replicates in groups from the 24 hr/RT and 72 hr/RT pairwise comparisons, suggesting that cold exposure induced strong and coordinated changes in expression of specific groups of proteins (Figure 2b, left-panel and middle-panel). Interestingly, UCP1 protein expression at 24 hr/RT showed higher variation among biological replicates than at 72 hr/RT, fitting with the assumption that some animals may exhibit decreased translational efficiency of this protein or that there is inter-individual variation in cold acclimation processes in scWAT during this early period. Moreover, the number of proteins uniquely expressed at 72 hr was 31.4% greater than the number of proteins differentially expressed at 24 hr vs RT (Figure 2a). The marked increase in protein changes after 72 hr cold challenge suggested a coordinated temporal modulation of protein expression that may be distinguishable between 24 hr and 72 hr – and may be an important window of tissue remodelling. Therefore, we investigated the variation in differential expression among biological replicates at 24 hr and 72 hr. Interestingly, we found that there was more variation in differential expression in our 72 hr vs 24 hr pairwise comparison among the top 20 DE proteins (Figure 2b, right-panel). These results indicated that the functional relevance of DE proteins likely overlap between 24 hr and 72 hr of cold exposure and may be variable in their temporal modulation. As expected, we found significant upregulation of UCP1 at both 24hrs and 72hrs. UCP1 is a canonical adipocyte browning marker that uses anionic fatty acids to facilitate uncoupling of the mitochondrial intermembrane proton-gradient to increase thermogenesis [59]. We also found a striking number of DE proteins related to cytoskeletal organization and endosomal formation/trafficking, which are key in cell polarity [60] and spatio-temporal signal regulation [61] (45.8%; 11/24 proteins excluding overlapping proteins).

Volcano plots provided an additional visual representation of DE protein distribution. We constructed volcano plots for each pairwise comparison and mapped the top 20 significant DE proteins (10 upregulated/10 downregulated) to each pairwise comparison (Figure 2c). We saw most DE proteins clustered near the threshold values (Figure 2c, dotted blue lines), clearly delineating a subset of DE proteins that may be suitable candidates for further characterization. Taken together, these results reinforced that adipose tissue dynamically responds to energetic needs, and that cold adaptation is a temporal response that likely coordinates multiple cellular processes – and likely multiple cell types – to facilitate a sufficient transition to thermogenic browning and enhanced sympathetic drive to the tissue.

### Several biological processes facilitate cold adaptation in a coordinated and time-dependent manner

**Figure 3.**
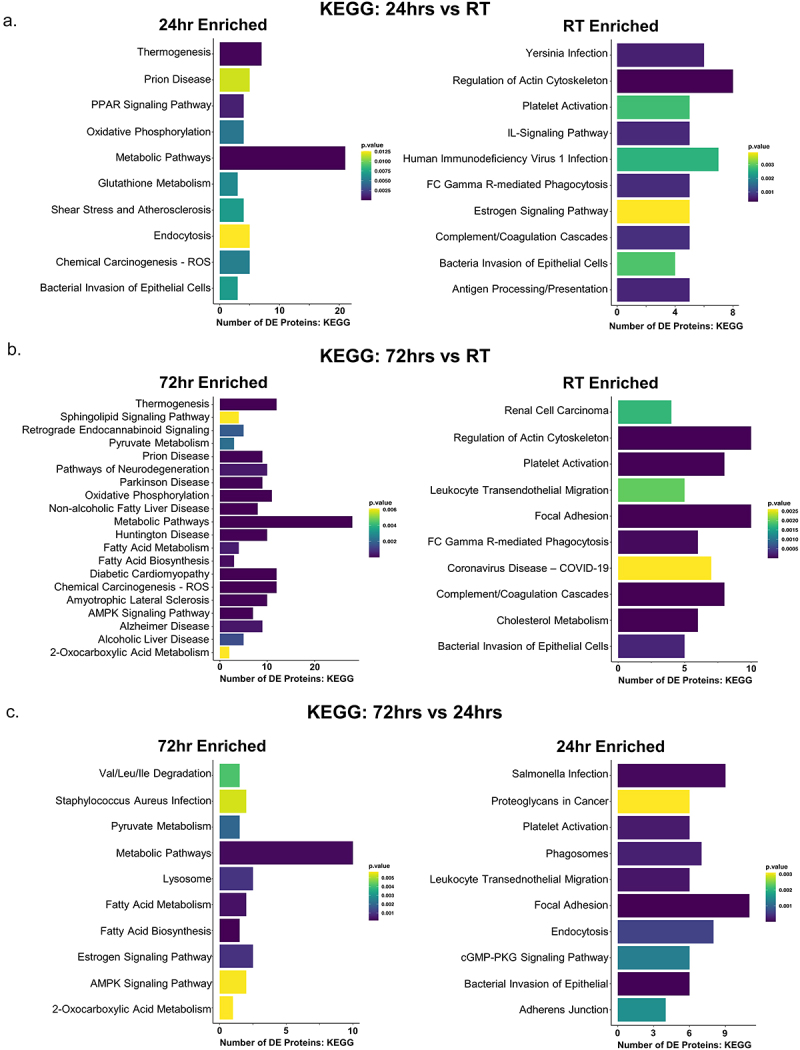
KEGG pathway enrichment highlights metabolic and innervation changes in scWAT following 24hr and 72hr cold exposure. Kyoto encyclopedia of genes and genomes (KEGG) analysis of three pairwise comparisons: (a) 24/RT, (b) 72h/rt, and (c) 72h/24h. Bar length represents the number of differentially expressed (DE) proteins found in each KEGG term. Bar color represents *p*-value, on a light (less significant) to dark (more significant) gradient.

**Figure 4.**
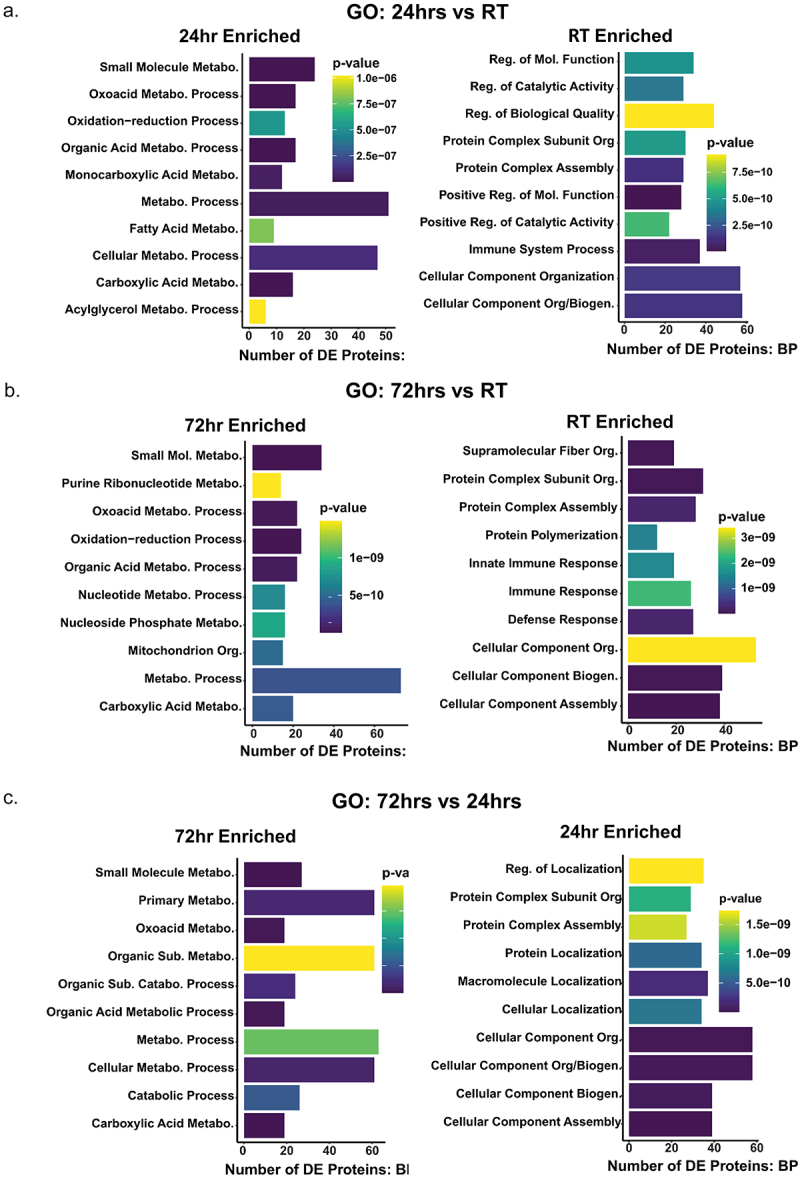
GO term enrichment highlights a wide range of modulated biological processes after 24hrs of cold and a transition to metabolic processes after 72hrs of cold exposure.Significantly enriched gene ontology (GO) terms for three pairwise comparisons in the biological process annotation: (a) 24/RT, (b) 72h/RT, and (c) 72h/24h. Bar length represents the number of differentially expressed (DE) proteins found in each GO term. Bar colour represents p-value, on a light (less significant) to dark (more significant) gradient.

Gene ontology (GO) terms and the Kyoto Encyclopedia of Genes and Genomes (KEGG) pathways provided a high-level and physiologically relevant view of biological outputs when viewing large datasets, such as proteomics. We used the limma package in R to identify enriched biological processes across our pairwise comparisons. We hypothesized there would be an enrichment in mitochondrial biogenesis and metabolic processes, as these are common reports from prior research literature. As expected, we found that the KEGG pathways enriched in cold groups (24 hr and 72 hr) were primarily metabolic pathways, but also included immune response and nerve-related pathways (Figure 3). Compared to RT, at 24 hr we found the strongest enrichment in metabolic and thermogenesis (Figure 3a), which persisted to the 72 hr time point (Figure 3b). Compared to RT, both 24 hr (Figures 3a, 4a) and 72 hr (Figures 3b, 4b) cold exposure showed enrichment in small molecule metabolism, metabolic processes, fatty acid metabolism, and oxidation-reduction, and at 72hrs there was significant enrichment in several additional metabolic processes (Figure 4b-c). At 72hrs of cold exposure we found specific enrichment of purine ribonucleotide metabolism and indications of reduced immune response (Figure 4b). This was a strong indication that our data were consistent with the literature, since purine nucleotides are known potent inhibitors of the thermogenic UCP1 [62–68]. To our knowledge, the data presented in this paper are the first evidence of a temporally driven switch to increase purine nucleotide metabolism and UCP1 expression simultaneously at this early time point. Interestingly, innate immune system processes were most enriched at RT compared to 24 hr cold (Figures 3a, 4a) or 72 hr cold exposure (Figures 3b, 4b). We also found significant enrichment in terms associated with cellular remodelling (Figure S2) and molecular function (Figure S3) at 24hrs cold exposure compared to RT, including increased mitochondria biogenesis, which persisted at 72hrs cold exposure (Figure S2). Together, these results suggested that changes to cellular organelles was initiated by 24hrs of cold exposure, and was coupled with a significant enhancement of metabolism across a variety of substrates (Figures 3–4).

### Proteomics pathway analysis

We next analysed our proteomics data using Ingenuity Pathway Analysis (IPA). IPA is a software platform developed by Qiagen that incorporates over 20 databases, as well as manually curated data, to determine several bioinformatic outputs [65]. As a resource, IPA provides information on Top Canonical Pathways, Predicted Upstream Regulators, and Diseases and Functions Networks. We focused our investigation on Top Canonical Pathways and Diseases and Functions Networks, based on their relatedness to the Biological Process category we used to analyse our results in the goana function of limma, as described above. Top Canonical Pathways between all pairwise comparisons were the same with the same number of molecules contributing to pathway predictions in pairwise comparisons (Suppl. Table ST6), however, predictions of whether they were activated or inhibited did vary by length of cold exposure (Figure 6). IPA also computed a pathway activity score called a Z-score where a positive Z-score indicates the pathway is predicted to be activated, while a negative Z-score is predicted as inhibited, and a Z-score of greater than 2 or less than −2 indicates a significant prediction. Therefore, we filtered all canonical pathways for the top 5 predicted activated (Suppl. Table ST7) and top 5 predicted inhibited (Suppl. Table ST8). Importantly, all pathways with significant Z-scores also had significant *p*-values (<0.05).

**Figure 5.**
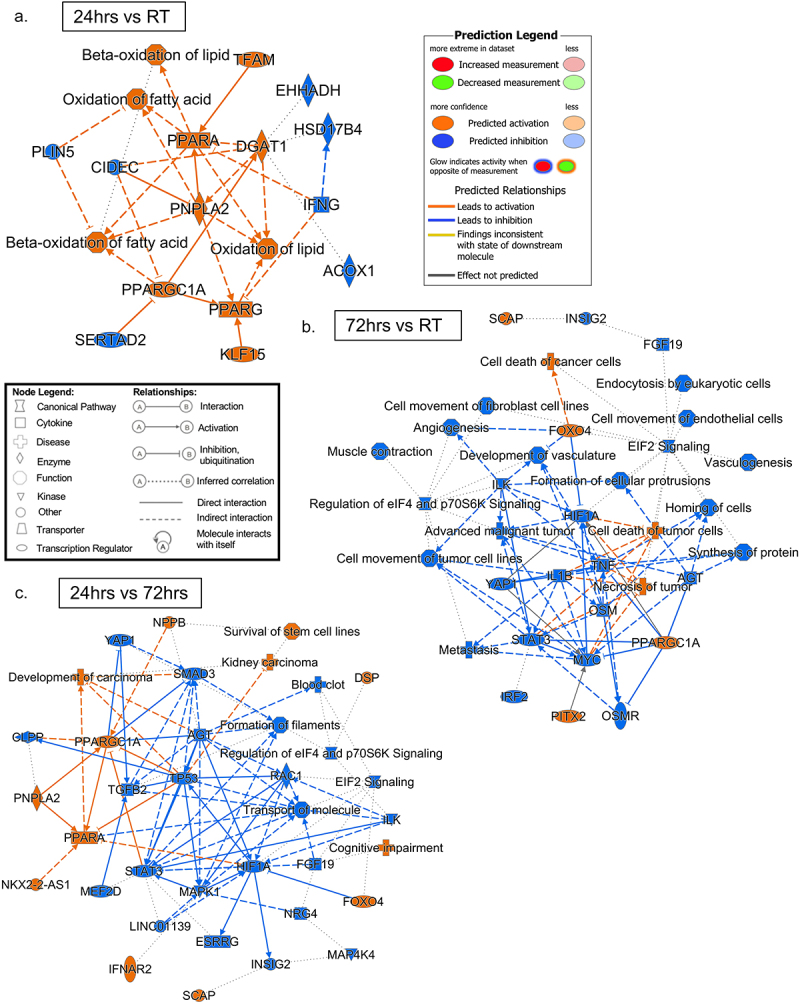
Ingenuity pathway analysis (IPA) graphical summaries for pairwise comparisons. Ingenuity pathway analysis (IPA) graphical summary of major biological themes for pairwise comparisons (a) 24/RT, (b) 72h/rt, and (c) 24h/72hr.

**Figure 6.**
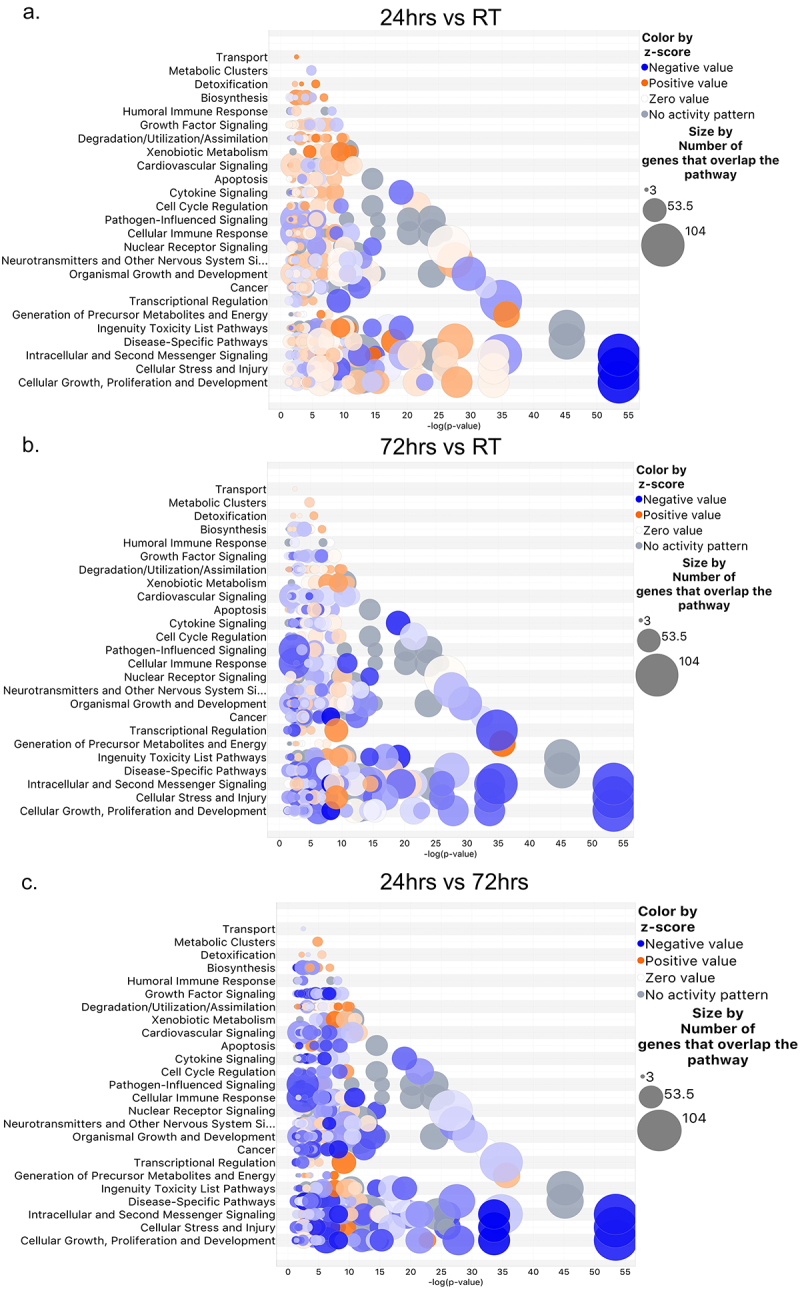
IPA pairwise comparison of canonical pathways. IPA generated top canonical pathways for each pairwise comparison (a) 24/RT, (b) 72h/rt, and (c) 24 hr/72 hr, with predicted activation pattern (colored by z-scores) and size representation of the number of molecules that overlap the pathway.

The major biological themes revealed by IPA are shown in Figures 5 and 6. The graphical summaries of pairwise comparisons are shown in Figure 5 and are represented as predicted canonical pathways, diseases, and functions that are either activated (orange) or inhibited (blue). During the first 24hrs of cold exposure there was more pathway activation, especially for metabolic pathways such as fatty acid utilization (Figure 5a). However, by 72hrs of cold exposure, more pathway inhibition becomes prevalent (Figure 5b-c). The one constant between RT and either time point of cold exposure was the maintained activation of the well-studied master transcriptional regulator PPARGC1A (PGC-1a), which was consistent with the expectation that adaptive thermogenesis and mitochondrial biogenesis would engage while the animal is in a continuous cold environment (Figure 5a-c). Interestingly, the same top pathways were impacted in all pairwise comparisons (Figure 6a-c), with similar numbers of DE genes contributing to pathway predictions. The differences were in which pathways were up- or down-regulated, depending on length of cold exposure. For example, while growth factor signalling was upregulated in the first 24 hr of cold exposure, it was down-regulated at 72 hr compared to RT (Figure 6a-b). IPA’s ‘Top Canonical Pathways’ showed reduced stress signalling (immune response -EIF2 signalling and Regulation of eIF4 and p70S6k Signalling) and enrichment of metabolic regulation (mitochondrial dysfunction, oxidative phosphorylation, and sirtuin signalling) (Figures 5–6). We compared these results to our KEGG analysis and found similar trends (Figure 3).

As expected, we also found enhanced oxidative phosphorylation and lysosomal clearance (CLEAR signalling pathway) [69] (Figure 3). Unexpectedly, we found enhanced apelin, a canonical promotor of angiogenesis and epithelial cell migration/proliferation [70] signalling in conjunction with angiogenic inhibition (Figure 5), while it is known that cold increases angiogenesis in scWAT [71]. While cold enhances angiogenesis in scWAT [71], the presence of angiogenic factors in scWAT is transient and adipose tissue angiogenesis is regulated by a balance of adipocyte-secreted pro- and anti-angiogenic factors [71]. Considering our data are looking at discreet time points we may only have captured time points of increased angiogenic inhibition to balance vessel expansion appropriately in the tissue, but this would need to be tested in separate studies. The dataset also revealed reduced fibrotic pathway expression (i.e. Wound Healing, LXR/RXR Activation, Pulmonary Fibrosis Idiopathic Signalling), reduced stress signalling (EIF2 and eIF4), and reduced proliferation (HER2 Signalling) [72] (Suppl. Table ST8), also fitting with some prior observations of WAT plasticity with cold.

In assessing IPA’s Top Network by Pairwise Comparison, with the top three canonical pathways overlaid on each other (Suppl. Fig. S4), we found that these data were not only consistent with our KEGG analyses, indicating pathway enrichment of neuronal processes at 72 hr cold exposure (Figure 3b-c), but they also revealed that nerve-related remodelling was occurring as early as 24hrs after cold exposure (as evidenced by the predicted increase in synaptogenesis signalling pathway; Suppl. Fig. S4A). At 24hrs versus 72hrs there was a predicted increase in axonal guidance signalling, suggesting neurite remodelling may begin after synaptogenesis signalling (Suppl. Fig. S4C).

Finally, we analysed the top Diseases and Functions (Suppl. Tables ST9 and ST10) in IPA. We followed the same approach as the top canonical pathway analysis, where we first filtered by positive and negative Z-scores and isolated the top predicted activated and inhibited diseases and functions. Importantly, this approach did not yield any non-significant (p-value >0.05) enriched pathways, and without initial filtering for Z-score, several pathways would be considered insignificantly activated or inhibited (Z-score > −2 and < 2). We found a striking enrichment of anti-cancer functions in both 24 hr/RT and 72 hr/RT pairwise comparisons. Interestingly, metabolic enrichment seemed to only be among the top 5 functions at 24 h, while after 72 h (compared to RT or 24 h) we saw emergence of ‘Neurological Disease – Cognitive Impairment’. Additionally, neurological disease, general cell signalling, protein degradation, and stem cell survival pathways were highlighted in our 72 hr/24 hr comparison (Suppl. Table ST9). In contrast to predicted activated diseases and functions, we found reductions in cell motility, inflammation, protein synthesis, and intracellular transport across all pairwise comparisons for predicted inhibited diseases and functions (Suppl. Table ST10). Overall, these results were consistent with our original KEGG analysis: we found the same major pathways to be engaged in both IPA and KEGG analyses, namely those related to metabolic processes, immune system response, and neuronal response (Figures 3, 6, Suppl. Fig. S4).

We can conclude that while various software platforms provide different perspectives and are variable in the terminology used, the outcomes remained consistent. Taken together, our total analyses indicated that the most significant changes to scWAT proteome early in cold exposure included enhanced metabolism, reduced inflammation, modulation of the nervous system, and increased cytoskeletal elements related to cell motility.

### Cold-induced mitochondrial remodeling is time-dependent

Based on our results generated from KEGG and IPA which implicated several metabolic, and particularly mitochondrial-mediated, pathways (Figure 3 and Suppl. Table ST6) during scWAT cold adaptation, we also investigated mitochondrial-specific proteins. To do this, we used Cytoscape to map mitochondrial proteins using the STRINGdb applet, and filtered our dataset using the COMPARTMENTS tool for proteins primarily localized to mitochondria (COMPARTMENTS score ≥ 4) [73]. We further filtered for proteins with a p-value of <0.05 from our differential expression analysis. Finally, we mapped protein–protein interactions using a degree-related hierarchical-clustering circular layout (Suppl. Figure S5a). This cluster analysis organizes proteins by their number of protein-protein interactions with the most interconnected protein at the 6 o’clock spot, or the bottom centre node of the circle. We found 21 mitochondrial proteins were upregulated and 13 proteins were downregulated in our 24 hr animals compared to RT (Suppl. Figure S5A top-panel), 51 proteins were upregulated and 13 were downregulated at 72 hr compared to RT (Suppl. Figure S5A middle-panel), and 38 upregulated proteins and 12 downregulated proteins at 72 hr compared to 24 hr (Suppl. Figure S5A bottom-panel). We found at both 24 hr and 72 hr that ubiquinol-cytochrome C reductase and Rieske Iron-Sulfur Polypeptide (UQCRFS1) were upregulated and served as the most interconnected node. UQCRFS1 is the penultimate step in complex III assembly of the electron transport chain and is responsible for transferring electrons from cytochrome b to cytochrome c1 [74]. Notably, many NADH dehydrogenase components of the electron transport chain (NDUFA10, NDUFS1, NDUFS2, NDUFB5, NDUFB6, NDUFA13, and NDUFA11) were only found significantly upregulated in our 72 hr/RT comparison while at 24 hr (24 hr/RT) we found NDUFB11 and NDUFV3, and between 24 hr and 72 hr (72 hr/24 hr) we found NDUFS1, NDUFV2, NDUFB9, NDUFA13, and NDUFA11. The differential upregulation of several components of NADH dehydrogenases suggests an increase in mitochondrial biogenesis, energy production, and thermogenesis, as we expected in cold treated animals. Additionally, we found that UCP1 was temporally driven in expression and was significantly differentially expressed from 24 hr to 72 hr (Suppl. Figure S5A bottom-panel). Thus, mitochondrial function is temporally regulated in a coordinated response to ambient cold in the scWAT depot.

### Cold exposure induces downregulation of cytoskeletal elements and polarity proteins

In our KEGG and IPA analyses, we found several instances of reduced cell motility and cellular polarity in cold exposed groups (Figure 3 and Suppl. Table ST10). We therefore conducted the same analysis in Cytoscape used to investigate mitochondrial proteins (Suppl. Figure S5A), and instead filtered for proteins in the cytoskeletal COMPARTMENT (Suppl.Figure S5B). We found relatively consistent downregulation: after 24 hr (24 hr/RT) we found 17 downregulated proteins and 6 upregulated proteins (Suppl. Figure S5B top-panel); after 72 hr (72 h/RT) we found 20 downregulated proteins and 6 upregulated proteins (Suppl. Figure S5B middle-panel); and finally, between 24 hr and 72 hr (72 h/24 h) we found 21 downregulated proteins and 7 upregulated proteins (Suppl. Figure S5B bottom-panel). These proteins are all related in some way to cellular motility, since cytoskeletal modulation is a hallmark of cell movement. However, we found various patterns of interest. First, the protein S100a8, a calcium-binding protein involved in immune cell chemotaxis [75] and formation of the Calprotectin complex [76], was downregulated as early as 24 hr and was *not* significantly downregulated between 24 hr and 72 hr (Figures 5a). Second, the master regulator of cell polarity and small GTPase, Cdc42 [77,78], was downregulated between 24 hr and 72 hr, indicating a minimum of a 24 hr delay in reduced cell polarity. Third, we found downregulation of the septins (SEPT6, SEPT7, and SEPT11), cytoskeletal filaments that provide a diffusion barrier during cell division, stabilize membrane curvature, and may restrict diffusion of membrane-bound proteins – facilitating polarity [79–81]. Downregulation of septins may serve as an indicator of reduced cell polarity both in cell motility and proliferation, especially when accompanied by the reduction in Cdc42. Finally, we found reduced RAN, a GTPase involved in nuclear transport, after 24 hr followed by reduced lamin-B 2 (LMNB2), an inner-nuclear lamin involved in nuclear envelope stability and acts as a linker between the nucleus and the cytoplasmic cytoskeleton [82]. These results suggest several aspects of the cytoskeleton are modulated including, spatial distribution of proteins and organelles, density, and polarity. Changes to these processes may indicate a preparation for browning and development of new brown adipocytes in the tissue, as well as changes to the neuronal inputs requiring cytoskeleton rearrangement.

### Cold exposure alters scWAT immune cell populations

As previously mentioned, the immune response proteome was significantly reduced in our cold exposed animals (Figures 3–6). Therefore, we again used Cytoscape to map differentially expressed proteins specific to the immune system via the STRINGdb function TISSUES [83], which is comparable to COMPARTMENTS described earlier, but used to classify broader biological processes. These maps provide an easily digestible dataset to better understand potential molecular pathways involved in downregulation of scWAT immune responses. We found 37 downregulated proteins and 6 upregulated proteins after 24 hr of cold (Suppl. Figure S6A top-panel), 32 downregulated proteins and 15 upregulated proteins after 72 hr of cold (Suppl. Figure S6A middle-panel), and 24 downregulated proteins and 20 upregulated proteins between 24 hr and 72 hr of cold exposure (Suppl. Figure S6A bottom-panel). Our results overlapped with decreased cell motility and the downregulation we saw among cytoskeletal proteins including Cdc42 (Suppl. Figure S5B and Suppl. Figure S6A). Surprisingly, we found that UCP1 was now included in the TISSUE search for ‘Immune System’ (Suppl. Figure S6A). While UCP1 is known as a canonical marker for browning of scWAT, there are several recent studies linking UCP1 to immune cell migration both in adipose tissue and in other tissues [84,85]. Additionally, it was revealed previously that UCP1-null mice had increased infiltration of innate immune cells [86].

### Cold induced nerve remodeling in scWAT begins at 24 hr of exposure

Cold-induced nerve remodelling in adipose has been previously reported [11,30,87], however, exactly when these changes begin and how long they are sustained remains unclear. We previously reported that axonal guidance signalling and synaptogenesis were top upregulated pathways in cold-induced neuroimmune cells (CINCs) recruited to adipose following 10 days of cold exposure [88]. Our current results (Figure 6, Suppl. S4) indicate that nerve-related changes in scWAT begin as early as 24 hr after cold exposure and may be mediated by other cell types in addition to, or before, CINCs are recruited to scWAT. Here, we found that by 24 hr following cold exposure numerous proteins related to pre-synaptic plasticity/strengthening were upregulated in scWAT. There were also notable increases in vesicle transport and docking (Ankfy, Ap2a1, VAMP3), axonal branching and ion channel ubiquination (Nedd4), and Ca^+^ channels (Atp2b1) (Suppl. Figure S4, Suppl. Figure S6B). By 72 hr after cold there was a significant upregulation of neurofilament proteins (TUBB3, Nefh), in addition to synaptic strengthening proteins (Suppl. Figure S4, Suppl. Figure S6B). These data reveal that in scWAT pre-synaptic potentiation occurs in as little as 24 hr after cold exposure, with axon outgrowth/branching occurring by 72 hr.

## Discussion

After several days of cold exposure, scWAT accommodates an increased need for lipid utilization and non-shivering thermogenesis via numerous plasticity-related changes: browning (development of inducible brown adipocytes with UCP1+ mitochondria), increased innervation and vascularization, and changes to immune cell populations and the cytoskeleton (reviewed in [11]. Few studies have investigated the proteome of scWAT under any conditions [55,89–91], and only one recent study by Rabiee et al. used proteomics to identify changes in protein expression during cold exposure in mice [91]. In their article, Rabiee et al. conducted a differential expression analysis during both acute (up to 24 hr) and extended (1 week minimum) cold exposure treatments. They uncovered a critical transcription factor, YBX1 that regulated browning initiation. However, our study contextualizes several aspects of cold adaptation from the acute (24 hr) stage of cold exposure to moderate (72 hr) cold exposure. Here, we used proteomics and comparative bioinformatic analyses to identify a proteomic landscape of mouse ing-scWAT between 24 hr and 72 hr of tissue remodelling and acclimatization to cold stimulation. Our results show that 24–72 hr of cold is a critical time point in cold adaptation for the ing-scWAT depot, revealing both known and novel pathway changes, and numerous that are transient and nature and likely critical for this early period of tissue adaptation to cold. During the 24 hr–72 hr cold transition we saw significant changes in metabolic programming (Figures 3c, 5 –6, Suppl. Fig. S5A), immune response (Figure 6, Suppl. Fig. S6A), and cell motility and cytoskeletal organization proteins (Figure 3c, Suppl. Fig. S5A). Additionally, we found modulation of the nervous system (Figure 3c, Suppl. Fig. S4, Suppl. Fig. S6B). Together, these results indicated that the ing-scWAT adapts to cold by coordinating the function and modulation of several cell and tissue types in a time-dependent fashion; invoking new pathways (i.e. nervous system modulation and supportive metabolic pathways – like purine nucleotide metabolism) and inhibiting other pathways such as immune cell mobilization and angiogenesis that may be detrimental to the immediate physiological needs of adaptation to cold exposure.

Most notably, we identified changes in pre-synaptic potentiation in as little as 24 hr after cold exposure – a standout discovery from this dataset. While literature has described changes in axon arborization and neurite density in scWAT with longer-term cold exposure (typically after 7–14 days is reported in the literature), the effects on pre-synaptic terminals have been completely overlooked. The question of whether true ‘synapses’ (or terminals, junctions) exist in adipose tissue is debatable, largely because clear evidence of post-synaptic specialization is lacking and most of the axons appear to have en passant varicosities that indicate release of nerve products that can diffuse to nearby cells along the length of the axons. Therefore, pre-synaptic structures involved in nerve product release (e.g. norepinephrine, neuropeptides) are present throughout scWAT. Our previous work demonstrated that parenchymal axons in scWAT contain synaptic vesicle protein markers that are associated presynaptic proteins at axonal varicosities [92]. Additionally, we identified specialized nerve-terminal structures, which we call the neuro-adipose nexus (NAN), which innervate individual adipocytes and based on markers appear to serve as sites for neurotransmitter release [11,93]. As of now, it is unclear whether changes in synaptic potentiation due to cold exposure occur uniformly across the entire tissue, are localized specifically to sites of tissue browning, are associated with blood vessels (which are highly innervated), include only changes to en passant varicosities, or impact number of NANs. This dataset demonstrates that changes in neurite density represent only one facet of cold-induced neuroplasticity within scWAT, with more mechanisms that remain to be investigated. Alterations at the pre-synaptic terminal appear to be crucial contributors that seemingly occur first, thus presenting an exciting opportunity for future research.

We do not want to overstate interpretation of our bioinformatic analyses, and it is important to recognize that several limitations of proteomics and bioinformatics exist in the absence of comprehensive cellular, molecular and physiological studies investigating specific functional outcomes behind these proteomic shifts. Label-free proteomics offers unbiased quantitative results that provide excellent relative information about the proteome, a more relevant omics data set than transcriptomics since mRNA levels can often be in misalignment with protein levels for the same gene. However, quantitation of label-free proteins with peak abundance methods requires imputation and post-processing that may not provide perfectly consistent bioinformatic analyses at the level of individual proteins.

Additionally, adipose tissue is heterogenous with numerous cell types and subtypes of adipocytes, and therefore this study lacks single cell or cell-type resolution. The data did benefit from multiple software platforms and a variety of analyses that together revealed overlapping and consistent gene ontologies, which indicated that our analysis approaches were replicable. Finally, we wish to acknowledge that interpretation of these data is difficult both at the computational level and the human-cognitive level. We do not claim to understand every aspect of our bioinformatic analysis and implications for tissue functions and physiology. Instead, we have dedicated significant efforts to provide a well annotated dataset and our informed interpretation of these data for the scientific community, which can be built upon and expanded in the future. For example, the next logical step would be to conduct a similar experiment that assesses phosphoproteomics to investigate changes in the activation state of these protein networks, in addition to peptidomics that could capture changes to transient, difficult to measure neuropeptides in the tissue. We hope these data will provide a usable resource for the development of new hypotheses and a starting point to develop new therapeutics to better understand, treat and prevent metabolic disease related to adipose tissue health and function.

## Materials and methods

### Lead contact

Further information and requests for resources and reagents should be directed to and will be fulfilled by the Lead Contact, Kristy L. Townsend (kristy.townsend@osumc.edu).

### Data and code availability

The authors confirm that the data supporting the findings of this study are available within the article and within supplemental spreadsheets. RAW mass spectrometry proteomics data reported in this paper has been deposited to PRIDE via massIVE and can be downloaded through the mass spectrometry interactive virtual environment (massIVe) FTP (http://massive.ucsd.edu/MSV000087516/) or the accession number (PXD026262) through PRIDE. Further, processed mzML, mztab and the search engine results file generated during the database search for this paper are also deposited with PXD026262. Cytoscape Clustify Summary Files and scripts used to process and analyse data in this paper have been deposited to Figshare: https://doi.org/10.6084/m9.figshare.27234348.v1.

### Animals and animal facility

11-week-old C57BL/6J male mice (Jackson Laboratory stock number 000664) were acclimated to our Small Animal Research Facility at the University of Maine for 1 week before the experiment start date. Animals were housed under standard climate-controlled conditions, with standard light/dark cycle (12:12 hr), and *ad libitum* access to standard chow and drinking water. Upon arrival, mice were placed into groups of 2–3 animals per cage and kept in the same cage group for the duration of the experiment. Bodyweight was used to equally distribute mice into the following groups: 1) Room temperature (*N* = 5), 2) 24-hr cold challenge (5°C), and 3) 72-hr cold challenge (5°C), (*N* = 6). For cold exposure mice were housed at 5°C (cold) in a controlled and monitored environmental diurnal chamber with standard light/dark cycle (12:12 hr), (Caron Products & Services Inc., Marietta, OH) with *ad libitum* access to food and water. The mean daily ambient temperature of our facility was 22°C, therefore room temperature animals were kept at an ambient temperature of 22°C. All mice were anesthetized by CO_2_ treatment. Whole subcutaneous white adipose tissue was surgically removed and immediately frozen in liquid nitrogen before transfer to long-term storage at −80°C.

### Ethical approval

Animal handling and all procedures were performed in accordance with the University of Maine’s and The Ohio State University’s Institutional Animal Care and Use Committee (IACUC), and in compliance with the guidelines of the PHS Policy on Humane Care and Use of Laboratory Animals, and Guide for the Care and Use of Laboratory Animals. This study was approved by the University of Maine’s IACUC, under protocol A2017-09-04.

### Sample preparation for LC-MS/MS

Mouse adipose tissue was homogenized with a Fisher Tissuemiser in 50 mm triethylammonium bicarbonate (Sigma) containing 6 M guanidine hydrochloride, 10 mm Tris(2-carboxyethyl) phosphine (EMD Millipore), and 40 mm 2-chloroacetamide (TCI America) at a ratio of 100 mg/2 mL buffer. After homogenization, solid sodium deoxycholate (Sigma) was added to a final concentration of 1%. Samples were then boiled at 95°C for 30 min to promote protein denaturation as well as reduction and alkylation of disulphide bonds. Gross debris was cleared by centrifugation at 4°C for 20 min at 15,000 × g. Samples were then frozen overnight at −20°C so the upper layer of fat could be extracted and discarded on the following day. The supernatant was subjected to methanol-chloroform precipitation to remove any remaining lipids. Protein concentrations were measured by BCA assay (Pierce) according to the manufacturer’s instructions. The proteins were digested first by LysC (Wako) at 25°C for 4 hr (1:100 enzyme:substrate) and then by trypsin (Pierce, 1:50) for 18 hr at 37°C. The resulting peptides were desalted with C18 TopTips (Glygen) and dried by vacuum concentration.

### LC-MS/MS analysis

Dried peptide samples were reconstituted in 0.1% FA in 5% ACN and maintained at 6°C in an autosampler until analysis. For the LC-MS/MS analysis, 500 ng of sample was directly injected into an UltiMate 3000 RSLCnano system (Thermo Scientific) coupled online to a Q Exactive HF-X Hybrid Quadrupole-Orbitrap mass spectrometer (Thermo Scientific). The analytical column was a 25 cm Acclaim PepMap 100 C18 column (75 µm ID, 2 µm particle size) maintained at 50°C. Mobile phase A was 0.1% FA in HPLC grade water, and B was 0.1% FA in 80% ACN. Peptides were chromatographically separated over the course of 90 min using linear gradients of 5% to 35% B in 80 min followed by 35% to 50% B in 10 min. Finally, the column was washed with 90% B for 6 min and re-equilibrated at 2% B for 10 min. The flow rate for all steps was 300 nL/min.

Peptides were ionized by nESI using a spray voltage of 2.0 kV and a capillary temperature of 250°C. The mass spectrometer was operated in the data dependent mode so that one MS1 scan was followed by HCD fragmentation (30 NCE) of the 15 most abundant ions. MS1 scans were done at 120,000 resolution (at m/z 200) from 350 to 1500 m/z (50 ms max IT, 3e6 AGC target). MS2 scans were acquired at 15,000 resolutions with an isolation window of 1.3 m/z, a max IT of 30 ms, an AGC target of 5e4, and a dynamic exclusion time of 60 s.

### Database searching

Mass spectra from all samples (*N* = 17) were converted to mzML with ProteoWizard [94] and OpenMS (v 2.5.0) [95]. Converted files were searched on the OpenMS platform with MSGF+ search engine and Biosaur feature detection [96] against a reviewed UniProt mouse proteome (downloaded 12/07/2020) containing the cRAP and MaxQuant contaminant FASTAs. Search parameters included: full trypsin digest, 1 missed cleavage, carbamidomethylation of cysteine as a fixed modification and oxidation of methionine as a variable modification with precursor and fragment mass tolerances of 20 ppm and 0.05 Da. PSM rescoring was performed with Percolator and protein inference was performed with Epifany across all samples, setting peptide and protein false discovery rates to 0.01.

### Proteomics data processing and differential expression analysis

All data analysis was performed with R (version 3.6.2) with various packages and is included in Supplemental Material (Suppl. Tables ST1-ST3). Samples were first grouped according to treatment: room temperature control (RT, *N* = 5), cold exposure for 24 hr (24 hr, *N* = 6) and cold exposure for 72 hr (72 hr, *N* = 6) and processed for differential expression analysis as described in Gardner et al., [57]. Briefly, samples were selected for pair-wise comparisons prior to filtering out lowly expressed proteins, and missing values were imputed with a multiple imputation approach by treatment group. Data was quantile normalized, and significance (p-value <0.05) determined by a modified exact test. Downstream gene ontology (GO), KEGG and pathway analyses utilized the list of significant proteins identified. For GO and KEGG, proteins were filtered by direction of the log fold-change to produce protein lists specific to that treatment. Mass spectrometry data is available through the mass spectrometry interactive virtual environment (massIVE) at the following hyperlink: MassIVE Dataset, or by searching the MassIVE database for MSV000087516, or by accession number PXD026262 through PRIDE.

### Proteomics data analysis using ingenuity pathway analysis (IPA)

Canonical pathway, disease, and functional analyses were generated using QIAGEN IPA (QIAGEN Inc., https://digitalinsights.qiagen.com/IPA) [97]. All differentially expressed proteins (including non-significantly differentially expressed) proteins were included in our IPA analysis. Parameters for IPA analysis were default parameters, with no restrictions. was performed from count, logFC and q-value from the unfiltered, differentially expressed dataset. For IPA analyses, a 5% FDR threshold was applied to DE genes and a Z score (−2.0 ≤ Z ≥ 2.0) was considered significant. Top 20 IPA derived canonical pathways sorted by significance were extracted from the IPA analysis and included in results. We then identified the top 5 most predicted activated (highest Z-score) and predicted inhibited (lowest Z-score) pathways that also maintained a p-value of <0.05. We followed top canonical pathway analysis by extracting the top 5 most predicted activated and predicted inhibited pathways from the ‘Diseases and Functions’ category. Again, we maintained a p-value <0.05 and generated protein networks to export all molecules included in the category. The top protein network for each pairwise comparison has generated and overlaid with top 3 canonical pathways for each pairwise comparison.

### Protein–protein interaction networks

All data were analysed for protein–protein interaction using Cytoscape (v 3.8.2) [98]. Protein interaction networks were generated with significant DE proteins (q-value <0.05) using the stringAPP applet. All protein interaction networks were constructed with a confidence value of 0.4 against the *Mus Musculus* genome. UniProt AC identifiers were used for all analyses. Proteins with COMPARTMENT scores of less than 4 were removed from mitochondrial interaction networks. Hierarchical circular layouts were generated using the clusterMaker tool and sorted by degree of interaction with the most interconnected node at the 6 o’clock position.

12-week-old C57BL/6 male mice were housed at room temperature (22°C) or cold (5°C) for 24 or 72 hr (Experimental Setup). Inguinal subcutaneous white adipose tissue (scWAT) depots were harvested and snap-frozen before processing (Tissue Harvest). Samples were processed as described in methods for LC-MS/MS analysis (Sample Processing). OpenMS and MSGet software platforms were used to identify and quantify proteins using peptide spectral data (peptide spectral graph; LC-MS/MS, Protein ID, and Quantification). A total of 2166 proteins were identified with considerable overlap among experimental and control groups (Venn diagram). Initial analyses of differentially expressed proteins revealed significant differences in protein expression between groups (volcano plot). Finally, functional groups of differentially expressed proteins were identified using a variety of functional annotation platforms and data analysis programs (R, Qiagen Ingenuity Pathway Analysis, and Cytoscape). In total, proteins involved in mitochondrial biogenesis, immune cell polarization, and nervous system modulation were most significantly altered by cold stimulation and likely are important for early tissue remodelling and browning.

## Supplementary Material

Supplemental Material

Blaszkiewicz_Johnson_2024_Suppl_FINAL.xlsx

## ARRIVE guidelines

We have adhered to ARRIVE guidelines for the reporting of this research.
