# Supplementary material for: The early transition to cold-induced browning in mouse subcutaneous white adipose tissue (scWAT) involves proteins related to nerve remodeling, cytoskeleton, mitochondria, and immune cells
Source: Adipocyte. 2024 Dec 6;13(1):2428938. doi: 10.1080/21623945.2024.2428938 (PMC11633174; doi:10.1080/21623945.2024.2428938)
Supplement: Supplemental Material [file KADI_A_2428938_SM3138.docx]

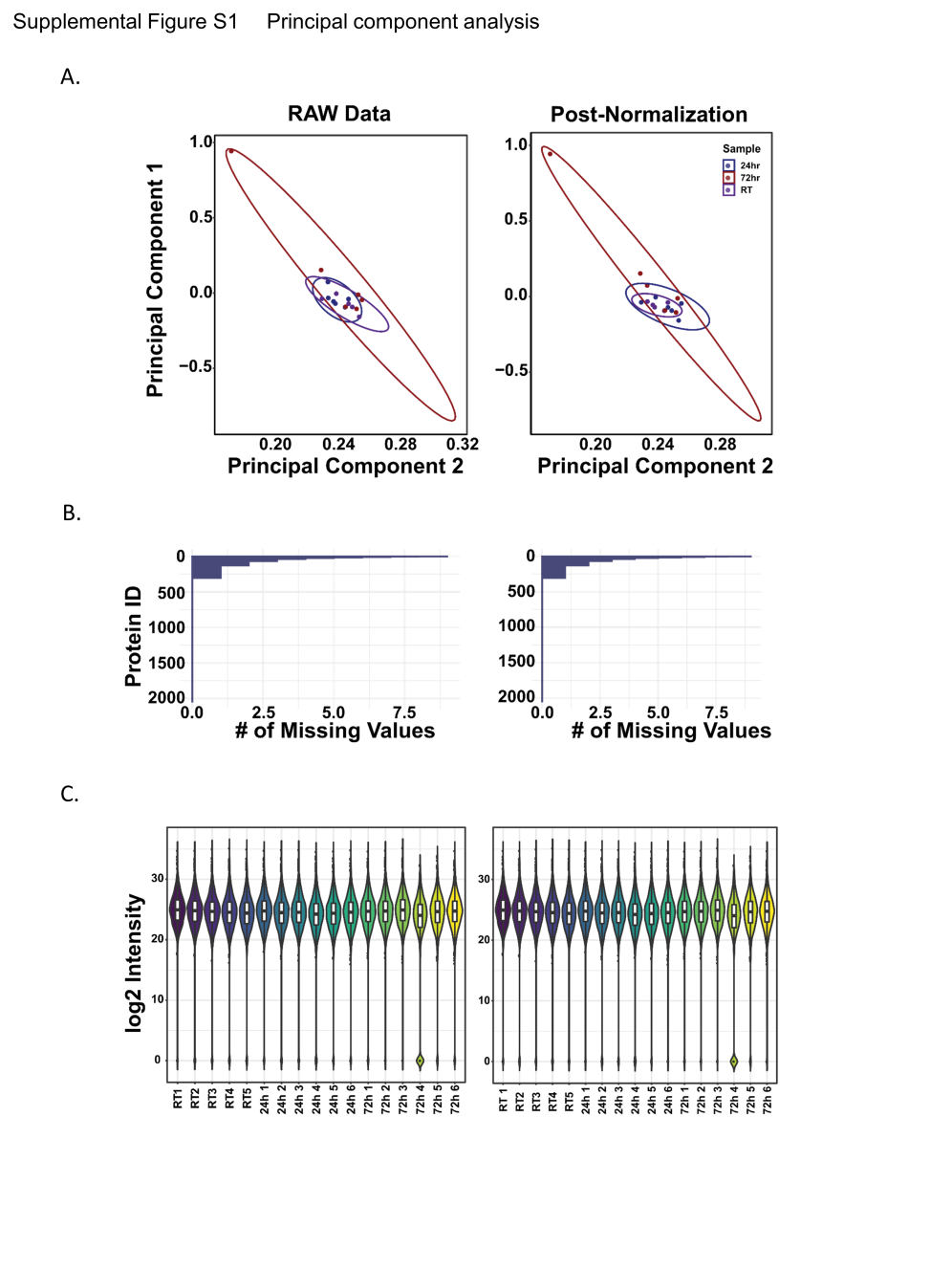


**Supplemental Figure S1: Principal component analysis.**

Principle component analysis (PCA) of principle components 1 (y-axis) and 2 (x-axis) from RT (purple), 24hr (blue), and 72hr (red) cold-treated animals showing pre-normalized (RAW) data (A, left-panel) and post-normalized (Post-Normalization) data (A, right-panel). Analysis of the number of missing values related to protein identification both RAW (B, left-panel) and post-normalized data (B, right-panel). Violin plots of the distribution of variance in Log2 Intensity values across all biological replicates in both RAW (C, left-panel) and post-normalized data (C, right-panel).

*Alt Text: Graphical depictions of the principle component analysis with and without normalization.*


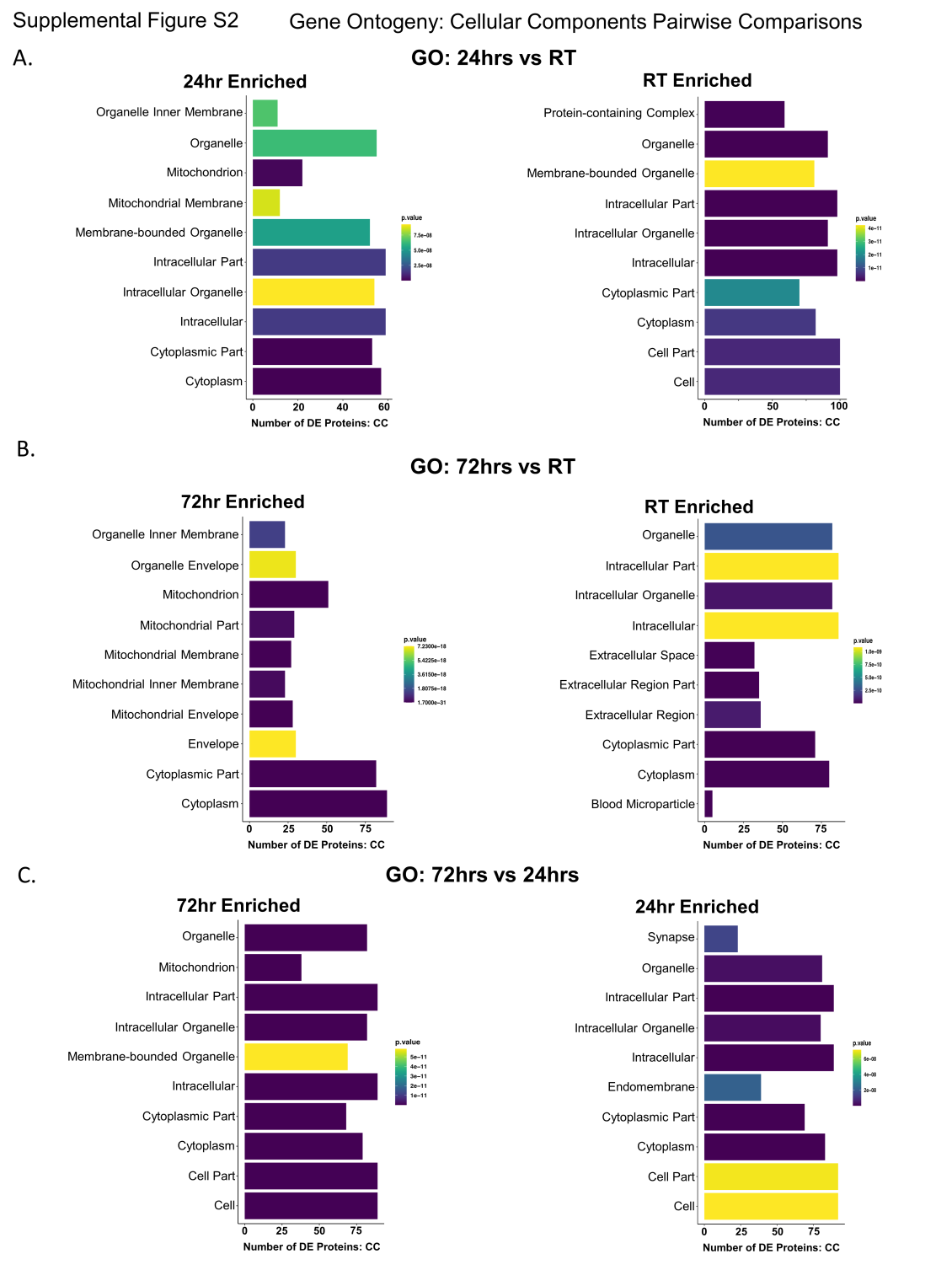


**Supplemental Figure S2: GO term enrichment highlights a wide range of changes in**

**cellular components 24hrs of cold with further changes evident after 72hrs of cold**

**exposure.**

Significantly enriched gene ontology (GO) terms for three pairwise comparisons in the cellular components annotation: (A) 24/RT, (B) 72h/RT, and (C) 72h/24h. Bar length represents the number of differentially expressed (DE) proteins found in each GO term. Bar color represents p-value, on a light (less significant) to dark (more significant) gradient.

*Alt Text: FigS2A, Gene ontogeny of biological processes enriched between 24-hours cold*

734 *exposure and room temperature. FigS2B, processes enriched between 72-hours cold exposure* 735 *and room temperature. FigS2C, processes enriched between 72-hours cold exposure and 24-*736 *hours cold exposure.*


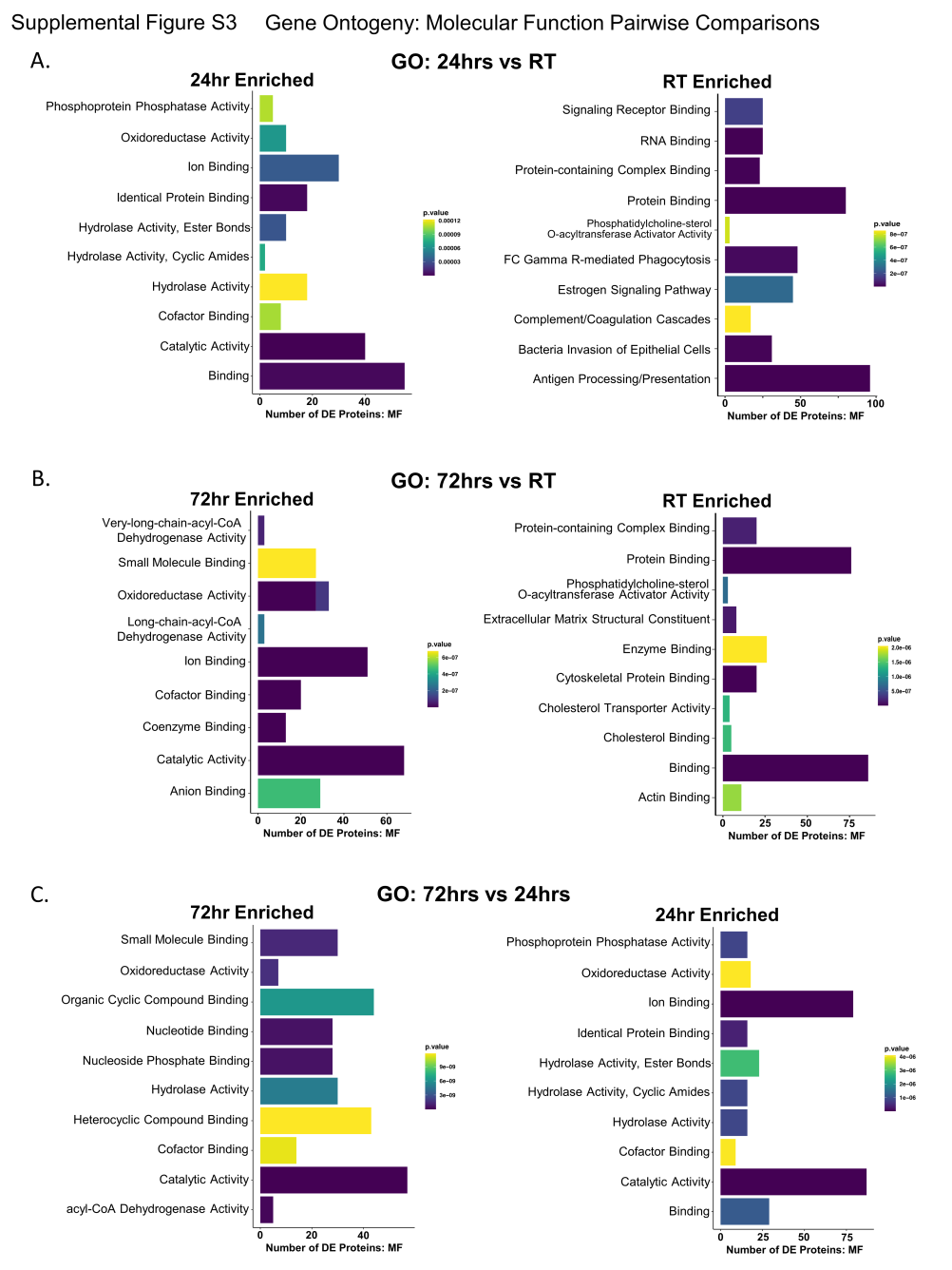


**Supplemental Figure S3: GO term enrichment changes in molecular function between**

**room temperature, 24hrs, and 72hrs of cold exposure.**

Significantly enriched gene ontology (GO) terms for three pairwise comparisons in the molecular function annotation: (A) 24/RT, (B) 72h/RT, and (C) 72h/24h. Bar length represents the number of differentially expressed (DE) proteins found in each GO term. Bar color represents p-value, on a light (less significant) to dark (more significant) gradient.

*Alt Text: FigS3A, Gene ontogeny of molecular functions enriched between 24-hours cold*

*exposure and room temperature. FigS3B, processes enriched between 72-hours cold exposureand room temperature. FigS3C, processes enriched between 72-hours cold exposure and 24-hours cold exposure.*


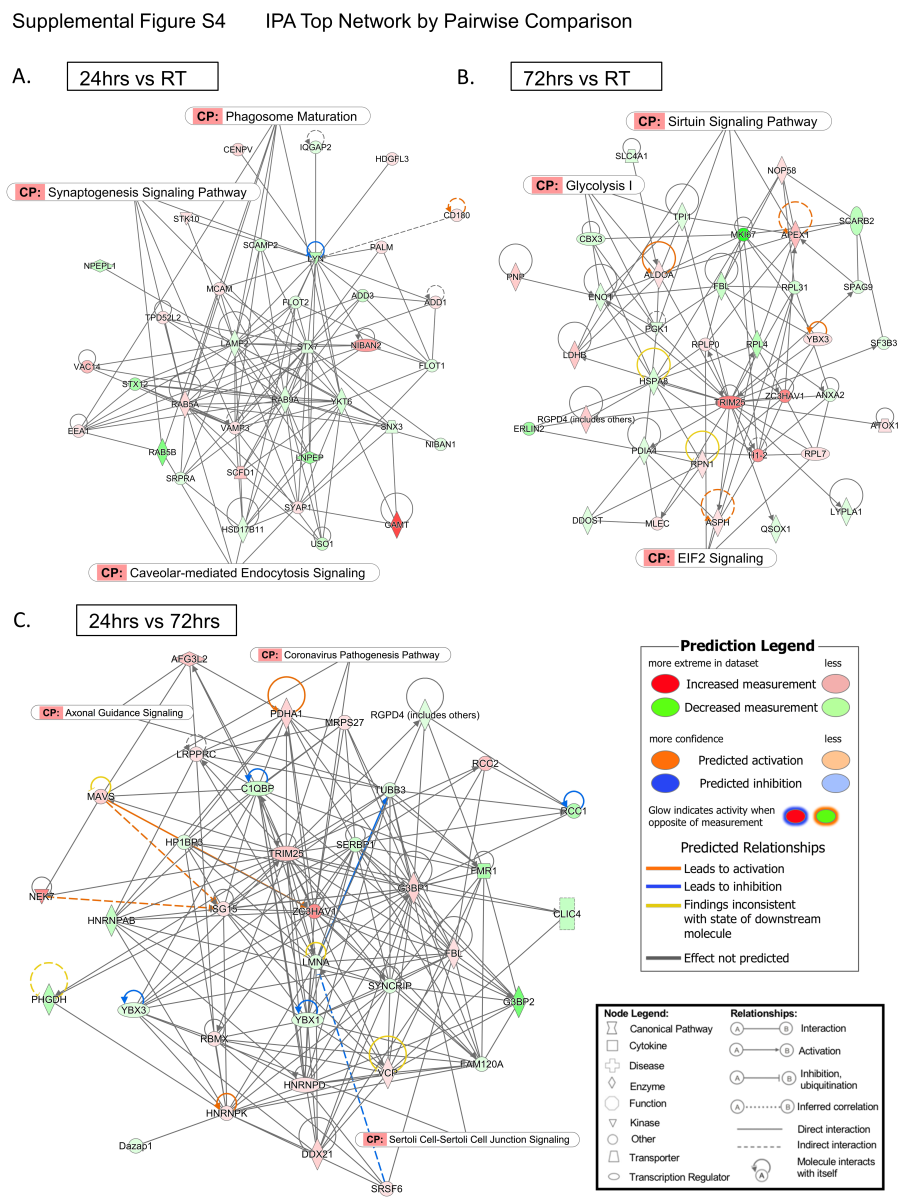


**Supplemental Figure S4: IPA top network by pairwise comparison.**

IPA generated top gene interaction network with top 3 Canonical pathways (CP) overlaid to show key molecules involved for each pairwise comparison(A) 24/RT, (B) 72h/RT, and (C) 24h/72.

*Alt Text: Comparison of top networks of Ingenuity pathway analysis. FigS4A, 24-hours cold*

*exposure compared to room temperature. FigS4B, 72-hours cold exposure compared to room temperature. FigS4C, 24-hours cold exposure compared to 72-hours cold exposure.*


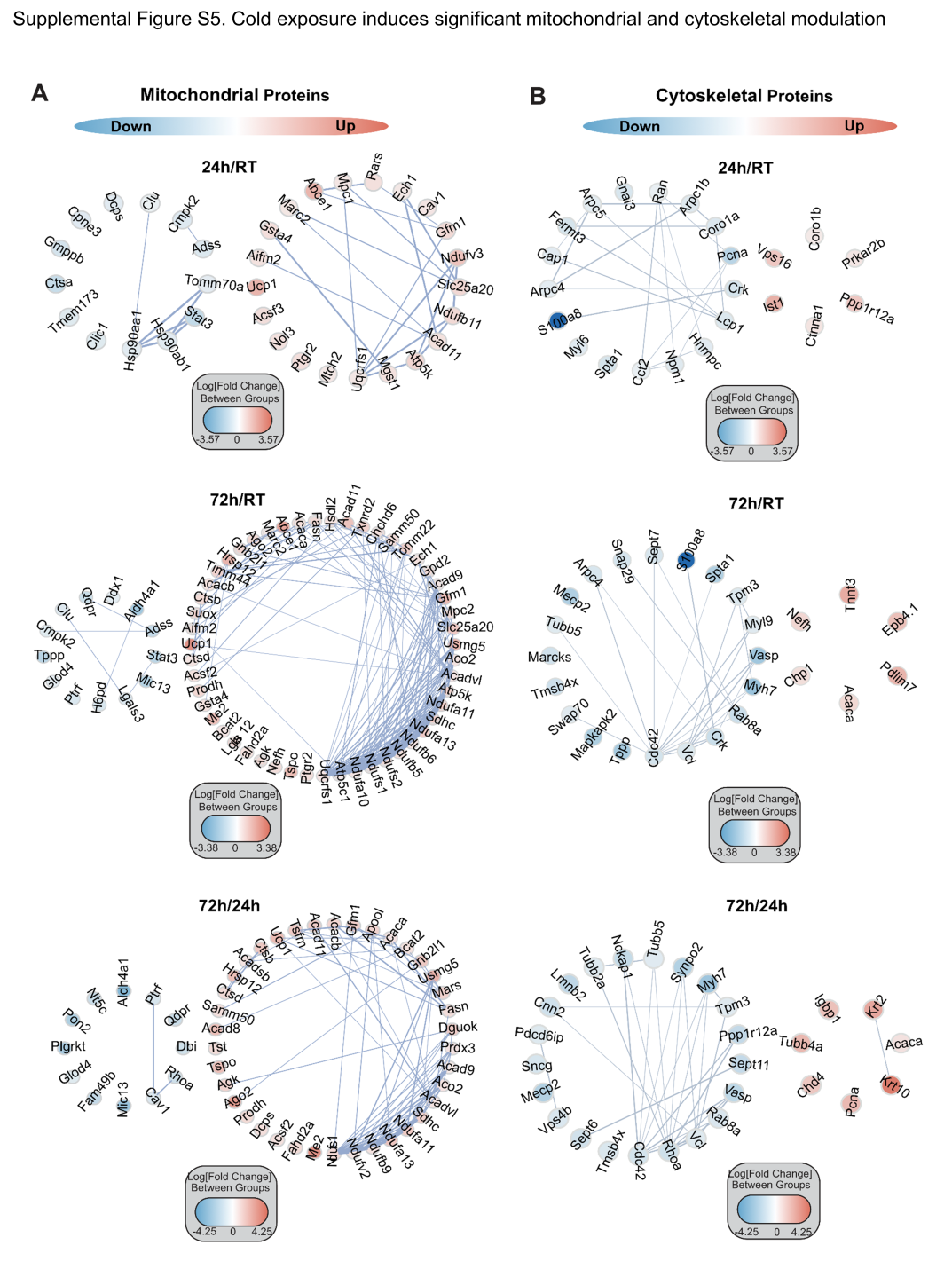


**Supplemental Figure S5: Cold exposure induces significant mitochondrial and**

**cytoskeletal modulation.**

(A) Differentially expressed mitochondrial proteins (nodes) and their protein-protein interactions (edges/lines) are in degree-sorted circular layouts 24h/RT (top-panel), 72h/RT (middle-panel), and 72h/24h (bottom-panel). Proteins were filtered for mitochondrial overlap using a COMPARTMENTS score ≥ 4. Node color saturation indicates level of significance

(light to dark; least significant to most significant respectively) in both upregulated (red) and

downregulated (blue) proteins. All proteins were filtered for a p-value ≤ 0.05 prior to the start of analysis. Proteins with the highest degree (greatest number) of protein-protein interactions (gray 779 edges) begins at 6:00 position and decreases moving counter-clockwise. (B) Differentially expressed cytoskeletal proteins mapped using the same parameters described in A but filtered for COMPARTMENTS score ≥ 4 under cytoskeleton.

782 *Alt Text: Up and down-regulated protein-protein interactions between room temperature, 24-hour*

*cold exposure, and 72-hour cold exposure. FigS5A, mitochondrial proteins. FigS5B, cytoskeletal proteins.*


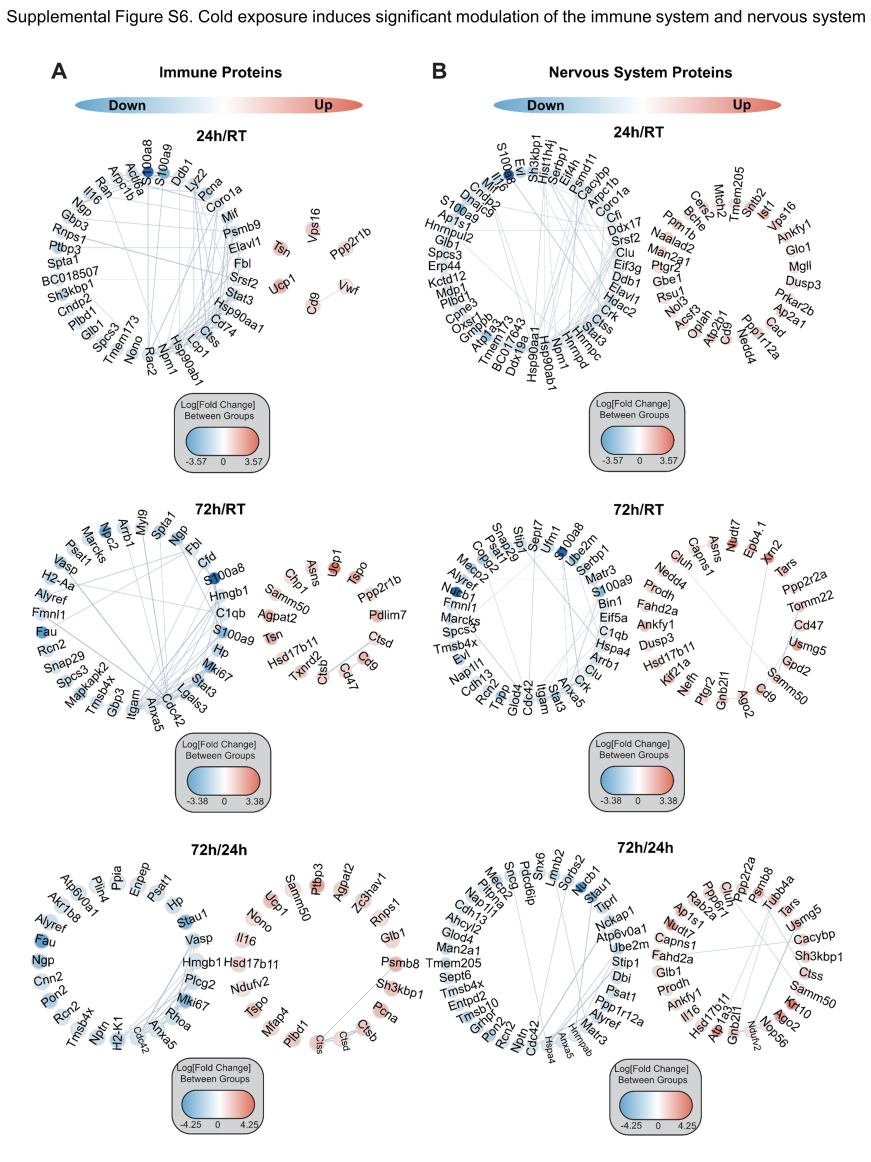


**Supplemental Figure S6: Cold exposure induces significant modulation of the immune system and nervous system.**

(A) Differentially expressed immune system proteins (nodes) and their protein-protein interactions (edges/lines) are in degree-sorted circular layouts 24h/RT (top-panel), 72h/RT (middle-panel), and 72h/24h (bottom-panel). Proteins were filtered for immune system overlap using a TISSUES score ≥ 4. Node color saturation indicates level of significance

(light to dark; least significant to most significant respectively) in both upregulated (red) and

downregulated (blue) proteins. All proteins were filtered for a p-value ≤ 0.05 prior to the start of analysis. Proteins with the highest degree (greatest number) of protein-protein interactions (gray edges) begins at 6:00 position and decreases moving counter-clockwise. (B) Differentially expressed nervous system proteins mapped using the same parameters described in A but filtered for TISSUES score ≥ 4 under nervous system.

*Alt Text: Up and down-regulated protein-protein interactions between room temperature, 24-hour cold exposure, and 72-hour cold exposure. FigS6A, immune proteins. FigS6B, nervous system proteins.*
